# Tautology explains evolution without variation and selection. A Comment on: ‘An evolutionary process without variation and selection’ (2021), by Gabora *et al.*

**DOI:** 10.1098/rsif.2023.0579

**Published:** 2024-09-18

**Authors:** István Zachar, Jakab Máté, Szabolcs Számadó

**Affiliations:** ^1^ HUN-REN Institute of Evolution, Centre for Ecological Research, Konkoly-Thege Miklós út 29-33, H-1121, Budapest, Hungary; ^2^ Department of Plant Systematics, Ecology and Theoretical Biology, Biology Institute, ELTE University, Pázmány P. sétány 1/C, Budapest, Hungary; ^3^ CSS-RECENS, Centre for Social Science, Tóth Kálmán u. 4, H-1097, Budapest, Hungary; ^4^ Department of Sociology and Communication, Budapest University of Technology and Economics, Egry János u. 1, H-1111, Budapest, Hungary

**Keywords:** evolution, population dynamics, percolation, Darwinian dynamics, selection

## Abstract

Gabora and Steel (Gabora L, Steel M. 2021 An evolutionary process without variation and selection. *J. R. Soc. Interface* 18, 20210334. [doi:10.1098/rsif.2021.0334]) claim that cumulative adaptive evolution is possible without natural selection, that is, without variation and competition. To support this claim, the authors modelled a theoretical process called self–other reorganization (SOR) that envisages a population of reflexively autocatalytic sets that can accumulate beneficial changes without any form of birth, death or selection, that is without population dynamics. The authors claim that despite being non-Darwinian, adaptive evolution happens in SOR, deeming it relevant to the origin of life and to cultural evolution. We analysed SOR and the claim that it implements evolution without variation and selection. We found that the authors, by design, ignore the growth and/or degradation of autocatalytic sets or their components, assuming all effects are beneficial and all entities in SOR are identical and immutable. We prove that due to these assumptions, SOR is a trivial model of horizontal percolation of beneficial effects over a static population. We implemented an extended model of SOR including more realistic assumptions to prove that accounting for any of the ignored processes inevitably leads to conventional Darwinian dynamics. Our analysis directly challenges the authors’ claims, revealing evidence of an overly fragile foundation. While the best-case scenario the authors incorrectly generalize from may be mathematically valid, stripping away their unrealistic assumptions reveals that SOR does not represent real entities (e.g. protocells) but rather models the triviality that fast horizontal diffusion of effects can effectively equalize a population. Adaptation in SOR is solely because the authors only consider beneficial effects. The omission of death/growth dynamics and maladaptive effects renders SOR unrealistic and its relevance to evolution, cultural or biological, questionable.

## 1. A misguided, non-evolutionary model

Gabora and Steel recently put forward the intriguing claim that cumulative adaptive evolution is possible *without* variation, competition and natural selection [1]. To support it, they have designed a theoretical process called self–other reorganization (SOR). SOR is a process akin to learning, involving hypothetical self-organizing and self-maintaining entities, but according to the authors, it differs from Darwinian evolution in that it lacks variation, selection, birth or death. Based on a mathematical model of SOR, they claim that it implements a cumulative evolutionary process of low fidelity, which might be relevant in modelling adaptation of non-Darwinian pre-biological or cultural systems.

However, according to our analysis, severe issues stem from the assumptions and misinterpretation of the SOR model by the authors, leading to flawed conclusions about the capabilities of SOR under any realistic condition. To assess the validity of our concerns, we have implemented an individual-based stochastic version of the SOR model, adding realistic assumptions and mechanisms that were ignored in the original article (see electronic supplementary material).

In SOR, the authors assume a population of entities housing reflexively autocatalytic and foodset-generated reaction networks (RAFs). They simplify RAFs by ignoring their chemical kinetics and molecular dynamics and only use them as placeholders for a dynamical entity that is in a steady state, and which can integrate new molecules to the reaction topology over time as external stimuli appear. The authors further assume that a population of identical RAF entities exist, separated in space, with no birth, death or splitting of entities and no (molecular) degradation. External stimuli may trigger the generation of new beneficial products in a population member, which ultimately percolate to every entity, causing thus community-based adaptation without reproduction, birth, death or competition, i.e. without Darwinian dynamics.

We found that SOR poses as a populational model with extensive horizontal exchange written in RAF language, while it actually ignores population (and internal) dynamics, competition and selection. The community-based model of SOR shows fast percolation over a set of non-replicating entities: a beneficial product gets fixed in the population faster (on average) than the next one appears. As such, their results are trivial, and SOR is not the first or the most sophisticated model of such horizontal exchange dynamics: there are many models (with or without population dynamics) that have already explored this simple process, such as the classical SIR infection model [2,3]. The SIR model assumes three types of individuals: susceptible (who may acquire the infection), infected (who got the infection) and recovered (who are cleared of the infection or are dead) and omits birth and death dynamics. SOR is practically the simplest case of SIR, assuming no recovery. We have shown in a multi-infectious SIR model that depending on how fast new infections (products in SOR terminology) appear, the population can be monomorphic (under ‘community-based evolution’) or polymorphic (under ‘individual-based evolution’) (see electronic supplementary material). Note that the successive infections in SIR cannot be considered a model of evolution by any means. While SOR is strikingly similar to SIR, neither SIR nor any of its extensions was acknowledged by the authors.

Moreover, the authors hard-coded into SOR the assumption that any change affecting the RAF entities is cumulative and beneficial, rendering their results inherently trivial and SOR to be irrelevant to any realistic context. However, there are even more fundamental issues with the approach the authors took. Gabora and Steel claim that ‘evolution is possible in the absence of variation and selection’ [1, p. 2]. This goes against a Darwinian interpretation of evolution (call it *evolution proper*), which is defined as the cumulative adaptive change of entities capable of multiplication, variation and heredity under selection [4–8]. For this claim to stand, the authors actually redefine ‘evolution’, hidden away in the electronic supplementary material, by removing any Darwinian aspect:

Evolution: descent with modification, or cumulative, adaptive change over time, giving rise to new species that share a common ancestor [3]. … Thus, here we use the more general definition of evolution used in cultural evolution research: cumulative, adaptive change over time (electronic supplementary material).

There is an overwhelming consensus that the theory of evolution by natural selection of replicating and competing entities capable of multiplication, heredity and variability is the adequate explanation for unsupervised adaptive change [4,6–10] supported by innumerable studies and experiments on long-term evolution and adaptation of various species [11–14]. The basic mechanism responsible for cultural evolution is similar according to many [4,15–19], as it shows the Darwinian principles. The difference is quantitative, depending on the amount of vertically inherited and laterally acquired information.

As a result, the authors commit circular reasoning. They claim that they have proven that cumulative evolution is possible without variation and selection. For this, they have redefined evolution *not* to involve variation and selection while modelling adaptive changes only. Hence, there is no need for variation and selection because, in SOR, every change is by definition adaptive. Thus, their claim becomes trivial, circular and insignificant. Especially, when one considers *evolution proper* in its biological sense. SOR does not implement *evolution proper*, while the article (foremost its title and abstract) misleadingly implies this, using ‘evolution’ without disclosing right away the authors’ meaning.

Without competition and selection, ‘adaptation’ in SOR does not make sense. The authors assume that entities receive only beneficial stimuli in this neutral tumbling. In fact, an RAF ‘tumbling down’ a sequence of truly random environmental stimuli, not only beneficial ones, would be as adaptive as a rock tumbling down a stream. In reality, random effects are more likely to be neutral or maladaptive and there is absolutely no guarantee that newly generated products of an RAF are beneficial. It would be like winning the lottery multiple times, sequentially. If there is no selection between the various products, maladaptive ones may spread equally likely. Thermodynamic effects could disrupt RAFs and entities. There is nothing that prevents this in SOR other than the authors’ baseline assumption to ignore maladaptive changes so that they can leave out selection from the model. We have modelled the effect of degradation and imperfect replication on SOR and our results support our claim that under realistic conditions (i.e. where not only beneficial changes can happen), SOR fails to accumulate adaptive changes (see electronic supplementary material).

Furthermore, we do not understand why it was important at all for Gabora and Steel to model a population of RAFs instead of a single RAF. A single RAF would react to stimuli and generate products the same way, without unnecessary percolation and population dynamics, which would make more sense in the prebiotic context. Adding a population seems only to serve the purpose that SOR becomes comparable to a conventional Darwinian model, *while population dynamics is ignored*. In reality (reflexively) autocatalytic sets have a tendency to grow (dynamically, not topology-wise), and they may grow exponentially: ‘AC reactions, cycles, and sets all have the potential for exponential growth of one or more of their products’ [20, p. 26].

While RAFs may theoretically exist in stable steady-state equilibrium [21], this equilibrium, in reality, should be extremely stable (both structurally and Lyapunov-wise) to withstand perturbations caused by stimuli and products (in reaction rates or concentrations). In the case where the equilibrium is not globally asymptotically stable, on one hand, short-lived perturbations may push the system to another fixed point, which is not necessarily stable, and which is differing from the equilibria of other entities in SOR, causing differences in the population, probably leading to competition for resources. On the other hand, if (membrane-bounded) RAFs grow autocatalytically, it should inevitably lead to cellular and populational dynamics inevitably leading to competition, birth and death, and differential survival or replication of individuals, i.e. to conventional Darwinian dynamics. These processes are entirely ignored in SOR to maintain the claim that it is a populational and evolutionary process without population dynamics.

**Figure 1.**
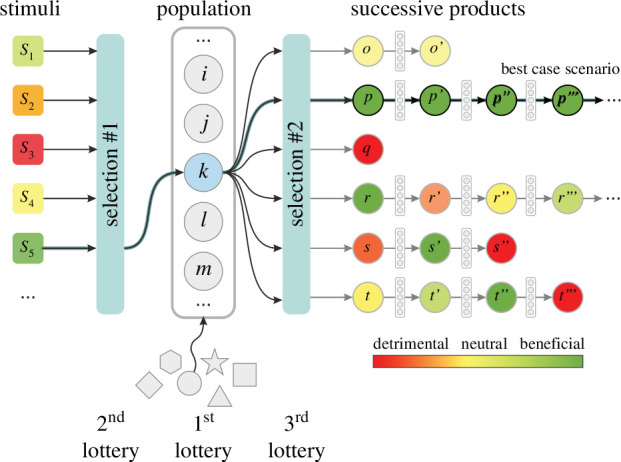
The triple lottery model of randomly accumulating changes within SOR, in our interpretation. An RAF of infinite adaptive potential is selected initially from many possible RAFs by unknown mechanisms (grey shapes, first lottery). A population of identical instances of this RAF (grey discs) are subject to stimuli $s_{i}$ (squares). Only beneficial stimuli can reach the population (second lottery). The different beneficial stimuli trigger the generation of different products (discs with index o,p,q,… ). Subsequent encounters with different RAFs in the population cause different lineages of subsequent products, e.g. p,p’,p’’,… [1] only consider adaptively beneficial products in SOR (third lottery) and ignore neutral or maladaptive ones (thick arrows). Here, we also include stimuli and products of non-beneficial effects (yellow and red boxes).

The authors assume that RAFs can autonomously and recursively ‘evolve’ ad infinitum*,* without selection or inheritance, when triggered with the right stimuli. The authors do not provide a mechanism for how such an RAF with infinite adaptive capacity (like a preformationist homunculus) emerges in the first place (lottery no. 1, see figure 1). There is an infinite number of different RAFs that can potentially exist, and out of these, only a few might have the potential envisioned by the authors. Simply picking the right RAF to present a best-case scenario is bad practice: one cannot generalize from it over a random initial RAF population.

Furthermore, the authors assume only beneficial environmental stimuli (lottery no. 2) and beneficial products (lottery no. 3), omitting maladaptive or downright detrimental effects. They ignore the thermodynamic necessities of real autocatalytic systems, which must maintain themselves (e.g. their steady state) against constantly appearing disruptive stimuli and products so that it is always true that all RAFs in SOR are identical. There is no selection or inheritance mechanism in SOR, hence the accumulation of beneficial adaptations can only happen because the authors assume that subsequent effects are always only increasingly beneficial without maladaptive effects or loss.

The authors incorrectly generalize from this triply best-case scenario. They assume that if it works by the mathematics, then the general case must also work under realistic conditions of the origin of life or cultural context, while they ignore realistic assumptions (e.g. degradation). Their selective assumptions exposed here clearly show how SOR is not a model of the origin of life entities. These, in turn, do not justify the characterization of SOR as a proper evolutionary or cumulative adaptation process, hence its biological or cultural relevance is questionable, at the least. Figure 1 illustrates SOR in our interpretation.

The authors set out early to support their model by providing a double strawman argument, staging a paradox (‘how organisms accumulate adaptive change despite that traits acquired over a lifetime are eliminated at the end of each generation’ [1, p. 1]) and claiming that Darwin’s theory was a response to this paradox. As a matter of fact, this paradox never existed, because Lamarck himself understood that not all changes accumulated during a lifetime are adaptive (e.g. loss of hair or teeth). As there is no guarantee that these changes are adaptive and there is no mechanism to prevent non-adaptive changes, no theory of adaptive evolution can be based purely on acquired changes.

Gabora and Steel claim that Darwin’s theory of natural selection is only applicable to systems where acquired changes are not inherited. According to them, whenever acquired changes are inherited (in Lamarckian systems), Darwin’s theory is insufficient.

This sequestering of germ cells from developmental changes is responsible for the *sine qua non* of a Darwinian process: lack of transmission of acquired traits … a set of coded self-assembly instructions used in these two distinct ways [copied during reproduction, interpreted during the development of the soma] that enables cumulative, adaptive change without transmission of acquired traits [1, p. 2]

This is misleading on two accounts: it falsely implies that (i) with two distinct roles of the instruction (copying the germline, developing the soma), adaptations are never the results of acquired traits and (ii) acquired traits only accumulate when there is no germline/soma distinction. Neither is true, as plants (lacking early sequestration of germline) attest [22]; however, the authors dispense away valid Darwinian explanations based on this claim. In fact, Darwin never proposed a strict separation of soma and germline. This was the idea of Weismann [23], to counter Lamarck [24]. Lamarckian and Weismannian inheritance can lead to Darwinian evolution as natural selection happens with both types of inheritance.

The key component (*sine qua non*) of Darwin’s theory is the inheritance of acquired mutations (changes) at the level of the genome, which leads to variation and natural selection. Mutations are de facto acquired during a lifetime, and without the inheritance of such acquired traits, no beneficial mutations would accumulate over time. If there is no soma/germline distinction, trivially *all* mutations of the DNA will accumulate over time, but this does not render the system less Darwinian. Cumulative adaptive Darwinian evolution happens with ‘nude’ replicators without a soma (Spiegelman’s experiments [25]). The point is, it is a quantitative matter in Darwinian inheritance how much information originates from the ancestral germline (genetic) and the soma (epigenetic) [26–28]. Hence, the author’s claim that the sequestering of the germline and the lack of transmission of acquired traits are what define Darwinian evolution, is simply wrong.

## 2. Summary

**Figure 2.**
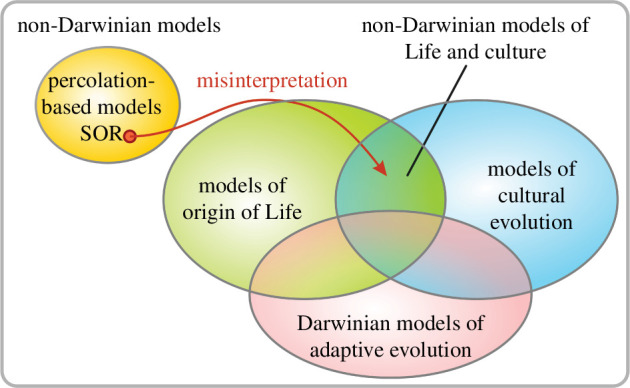
The misinterpretation of the SOR model by the original authors.

The authors are correct about their model being non-Darwinian. There is no competition, selection, reproduction and no change of entities or inheritance of their properties between generations (see figure 2). Based on our analysis, we go further in characterizing SOR: *it is a non-Darwinian, non-adaptive and non-evolutionary model of percolation over a static population*, akin to the well-known SIR model. The selection of unrealistic assumptions and the redefinition of ‘evolution’ by the authors only serve to pose their mathematical urn model (SOR) as a population-based evolutionary model. However, any accumulation of ‘adaptive’ changes in SOR happens only due to the fact that the authors ignore maladaptive effects, rendering SOR entirely disjoint from the real world. Their conclusions are the direct results of this tautology: SOR does not need the classical Darwinian variation–selection mechanism to generate ‘adaptations’ because by default all changes are beneficial (‘adaptive’); however, there is no selection to explain why there are no maladaptive products. The triviality of the result is not explained to the reader, and it is masked by the complexity of SOR’s formalism and the misleading use of the word ‘evolution’. In reality, without selection, there is no guarantee that maladaptive changes do not disrupt population members (RAFs), ultimately leading to the collapse of the population. Contrary to Gabora and Steel, we do not think that cumulative adaptive change could happen in nature without variation and selection of a population of replicators. Understanding the non-Darwinian processes that took place before the first true replicators and life on Earth have emerged is a central problem of origin of life research. Unfortunately, the scenario put forward by Gabora and Steel fails to adequately capture basic chemical principles, like molecular deterioration, and thus their model is not relevant to the field. Any single one of the issues we have raised invalidates the conclusions of their article. They practically recreated Hoyle’s fallacy: a tornado sweeping through a junkyard randomly assembles a Boeing 747, but in this case purely because they assume that only adaptive steps can happen.

## Data Availability

All source code required to replicate the results and figures are publicly available at Zenodo [29]. Supplementary material is available online [30].
